# Isolation of Zika Virus Imported from Tonga into Australia

**DOI:** 10.1371/currents.outbreaks.849adc0ad16beec4536695281707f785

**Published:** 2016-09-07

**Authors:** Alyssa T. Pyke, Peter R. Moore, Sonja Hall-Mendelin, Jamie L. McMahon, Bruce J Harrower, Tanya R Constantino, Andrew F van den Hurk

**Affiliations:** Public Health Virology Laboratory, Forensic and Scientific Services, Coopers Plains, Queensland, Australia; Public Health Virology Laboratory, Forensic and Scientific Services, Coopers Plains, Queensland, Australia; Public Health Virology Laboratory, Forensic and Scientific Services, Coopers Plains, Queensland, Australia; Public Health Virology Laboratory, Forensic and Scientific Services, Coopers Plains, Queensland, Australia; Public Health Virology Laboratory, Forensic and Scientific Services, Coopers Plains, Queensland, Australia; Public Health Virology Laboratory, Forensic and Scientific Services, Coopers Plains, Queensland, Australia; Public Health Virology Laboratory, Forensic and Scientific Services, Coopers Plains, Queensland, Australia

**Keywords:** Zika

## Abstract

Introduction: The globally emergent *Zika virus* (ZIKV) is a threat to Australia, given the number of imported cases from epidemic regions and the presence of competent mosquito vectors. We report the isolation of ZIKV from a female traveler who recently returned from Tonga to Brisbane, Queensland, Australia in 2016.

Methods: A specific TaqMan real-time reverse transcriptase polymerase chain reaction assay (RT-PCR) assay was used to detect ZIKV in serum and urine samples. Conventional cell culture techniques and suckling mice were employed in an attempt to isolate ZIKV from serum and urine.

Results: A ZIKV isolate (TS17-2016) was recovered from the serum sample after one passage in suckling mouse brains and harvested 11 days post inoculation. Phylogenetic analysis of complete envelope (E) gene sequences demonstrated TS17-2016 shared 99.9% nucleotide identity with other contemporary sequences from Tonga 2016, Brazil 2015 and French Polynesia 2013 within the Asian lineage.

Discussion: This is the first known report of successful isolation of ZIKV from a human clinical sample in Australia and the first from a traveler from Tonga. This study highlights the potential difficulties in isolating ZIKV from acute clinical samples using conventional cell culture techniques, particularly in non-endemic countries like Australia where access to samples of sufficient viral load is limited. The successful isolation of TS17-2016 will be essential for continued investigations of ZIKV transmission and pathogenicity and will enable the advancement of new preventative control measures extremely relevant to the Australian and Pacific region.

## Introduction

*Zika virus* (ZIKV) is an emerging mosquito-borne virus (arbovirus). Discovered in Uganda, Africa, in 1947^1^, ZIKV remained in relative obscurity for years until it caused explosive outbreaks on Yap, Federated States of Micronesia, in 2007 and in French Polynesia in 2013^2^^,^ 3. These Pacific outbreaks signified the first major epidemics of ZIKV disease, and collectively resulted in over 35,000 clinical cases^2^. Ongoing ZIKV transmission and spread has facilitated outbreaks in a number of Pacific island countries, including Tonga in 2016. Emergence of ZIKV in the Pacific led to its introduction into Brazil in 2015 and has increased the risk of local transmission in several countries, including Australia, via repeated importation from viremic travelers^2^^,^ 4.

ZIKV is a member of the genus *Flavivirus* and urban transmission is predominantly driven by *Aedes aegypti* 2 ^,^ 4. Locally acquired ZIKV infections in Australia have not been reported, however, endemic populations of *Ae. aegypti* constantly warrants rigorous disease surveillance and mosquito control strategies^4^. Indeed, since 2012, 68 cases of imported ZIKV infection have been reported in Australia up until August 2016 (http://www.health.gov.au/internet/main/publishing.nsf/Content/ohp-zika-notifications.htm). Concern also exists because recent evidence has implicated ZIKV infection in severe neurological disorders such as microcephaly in newborns and Guillain-Barré syndrome^2^.

Evolving from its origins in Africa, major East and West African and Asian ZIKV phylogenetic lineages have been defined. To date, all reported Pacific and American ZIKV isolates belong to the Asian lineage^5^. ZIKV has been isolated from non-human primate serum, mosquitoes and a range of human samples, including serum, urine, semen, saliva and breastmilk, albeit with limited success. Most reported isolates have been derived following intracerebral suckling mouse brain (SMB) inoculations or infection of African green monkey Vero and Vero E6 or *Ae. albopictus* C6/36 cell monolayers^2^^,^ 6 ^,^ 7. In this report, we describe the first Australian isolation of ZIKV from a clinical patient, a traveler from Tonga.

## Methods

In February, 2016, a female patient in her 30’s returned from Tonga to Brisbane, Australia with a fever and maculopapular rash (ProMED-Mail, *Zika Virus* (28), Archive Number: 20160524.4240474, http://beta.promedmail.org/post/4240474). An acute phase serum and a subsequent urine sample were collected 4 days apart. Both samples demonstrated ZIKV RNA by TaqMan reverse transcription PCR (RT-PCR)^4^ with cycle threshold (C_t_) values of 29 (serum) and 34 (urine). The RT-PCR ZIKV positive serum sample was negative for serology using a pan-flavivirus IgM microsphere immunoassay and a pan-flavivirus IgG enzyme linked immunosorbent assay. For virus isolation, each sample was inoculated separately onto C6/36 (ATCC, CRL-1660) and Vero (ATCC, CCL-81) cell monolayers and intracerebrally into 1-2 day old Swiss outbred suckling mice. Cells were incubated for 10 days at 28^o^C (C6/36) or 37^o^C (Vero) and cultures passaged 3 times. Cultures were monitored for virus growth using an immunofluorescence assay^8^. Mice remained asymptomatic and were euthanized to prepare SMB pools 11 days post-inoculation. SMB pools were screened for ZIKV RNA by RT-PCR.

Viral RNA extraction, RT-PCR and sequencing of complete ZIKV envelope (E) genes were performed as previously described^9^^,^ 10 using two overlapping hemi-nested RT-PCRs, ZIKV (A) - round 1 and round 2 and ZIKV (B) - round 1 and round 2 with the following primers: ZIKV (A) round 1, 627 For primer 5’-^627^AATGCCCTATGCTGGATGAG^646^-3’ and 1652 Rev primer 5’-^1652^GCCAAGGTAATGGAATGTCG^1633^-3’, ZIKV (A) round 2, 723 For primer 5’-^723^AAAAAGGTGAAGCACGGAGA^742^-3’ and 1652 Rev primer, ZIKV (B) round 1, ZIKV E For primer 5’-^1328^AAGTTTGCATGCTCCAAGAAAAT^1350^-3’^4^ and 2661 Rev primer 5’-^2661^GAGATCCCGCAGATACCATC^2642^-3’ and ZIKV (B) round 2, ZIKV E For primer and 2627 Rev primer 5’-^2627^TGACTGCTGCTGCCAATCTA^2608^-3’. Amplified DNA products from round 1 were diluted 1:100 and 5 µL added to round 2 mixes in a total of 50 µL. Alternatively, ZIKV (A) and ZIKV (B) RT-PCRs could be used as single round overlapping RT-PCRs when viral RNA levels were adequate. Amplification conditions used the following conditions: one cycle of 50^o^C for 15 min and 94^o^C for 2 min; 40 cycles of 94^o^C for 15 sec, 55^o^C for 30 sec and 68^o^C for 1 min followed by a final extension of 68^o^C for 5 min. Sequencing of the complete E gene was performed using the same amplification products and additional sequence primers: 1175 Rev primer 5’- ^1175^CCGATATTGATGCCTCATAG^1156^-3’, 1908 Rev primer 5’-^1908^GCTGCGGTACACAAGGAGTATG^1887^-3’, 2059 For primer 5’-^2059^TAACCCTGTAATCACTGAAA^2078^-3’ and 2379 Rev primer 5’-^2379^ATGAGAATTTGTGAGAACCA^2360^. Sequence positions for both the RT-PCR and sequencing primers were based on ZIKV Puerto Rico 2015, GenBank accession number KU501215.

Complete ZIKV E gene sequence (1512bp) alignments were performed using Clustal W and Mega 7.0 software^11^ and a maximum likelihood tree was inferred from the TS17-2016 sequence, additional sequences from ZIKV RT-PCR positive viremic travelers who had returned to Australia and available sequences on GenBank.

## Ethics Statement

This study and case reporting was approved by the Forensic and Scientific Services Human Ethics Committee.

## Results

Of all isolation attempts, only the serum-derived SMB was positive, with a C_t_ value of 29. To further amplify viable virus, the serum-derived SMB was then inoculated in duplicate onto C6/36 cells and intrathoracically into pools of ≤10 *Ae. aegypti* mosquitoes. Cell and supernatant fractions from cultures and mosquitoes were individually screened by RT-PCR. Mean±SD C_t_ values for the culture samples were 16.13±0.74 and 18.76±0.62 for the cells and supernatant, respectively, and a mean±SD C_t_ of 18.22±1.27 was obtained from 25 pools of positive *Ae. aegypti*.

The SMB-recovered ZIKV isolate TS17-2016 was passaged in C6/36 cells 4 times yielding 50% tissue culture infectious dose (TCID_50_) viral titers of 6.80, 9.15, 9.80 and 8.00 log_10_ TCID_50_/mL for passages 1, 2, 3 and 4, respectively. The mean±SD titer of the positive mosquito pools was 3.47±0.67 log_10_ TCID_50_/mL. The SMB viral titers were below the TCID_50_ assay threshold of detection.

Complete E gene sequences were determined for the TS17-2016 isolate and other strains imported recently into Australia via viremic travelers (GenBank accession numbers KX216632, KX216633, KX216634, KX216636, KX216637, KX216638, KX216639, KX380262 and KX380263). Phylogenetic analysis (Fig. 1) of the TS17-2016 ZIKV isolate (GenBank accession number KX216635) demonstrated the virus belonged to the Asian lineage together with contemporary Pacific and American strains and shared 99.9% nucleotide identity with sequences from Tonga 2016, Brazil 2015 and French Polynesia 2013 (GenBank accession numbers KX216634, KX280026 and KJ776791, respectively).

**Phylogenetic Tree:**
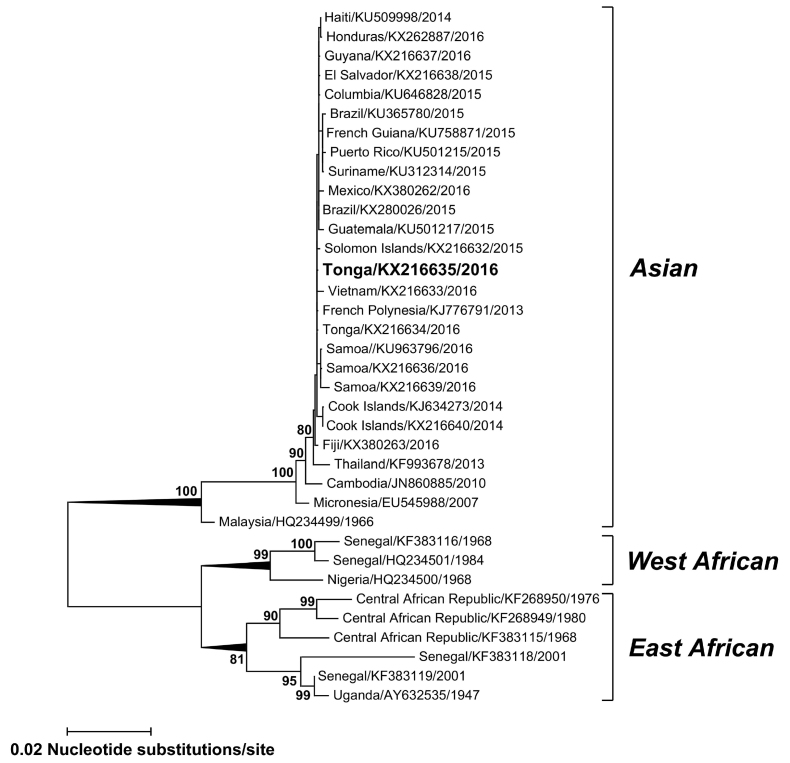
Maximum likelihood phylogenetic tree based on complete E gene nucleic acid sequences (1512 bp) constructed using MEGA 7.0^11^ with bootstrap support (1,000 replications). GenBank accession numbers are given for the ZIKV isolate TS17-2016 (KX216635) and other representative ZIKV global strains which group into one of the major Asian, East or West African lineages. Horizontal branch lengths are proportional to the bar representing the number of nucleotide substitutions/site. Percentage bootstrap support values are shown for key nodes.

## Discussion

This is the first known isolation and characterization of ZIKV from a clinical patient from Tonga and the first of this virus in Australia. Although local ZIKV transmission has not yet been detected in Australia, endemic populations of *Ae. aegypti* are present in northern Queensland, a popular tourist destination for overseas travelers, underscoring the potential risk of ZIKV introduction and transmission currently threatening this region. Recent vector competence experiments demonstrated that of 7 common Australian mosquito species tested, only* Ae. aegypti* could transmit ZIKV (Hall-Mendelin, S. *et. al*., 2016, Vector competence of Austalian mosquitoes for Zika virus, in press)^13^. However, the distribution of this species is restricted to northern Queensland and it is primarily this region that is receptive to local transmission of ZIKV.

In this study, the success of SMB inoculation and failure to recover live virus directly from patient samples using conventional cell culture techniques highlights potential difficulties in ZIKV isolation. Previous intracerebral inoculations of mice with ZIKV have resulted in symptomatic disease 6-10 days post inoculation^1^^,^ 14. In our study, mice remained asymptomatic for 11 days which could reflect a potentially low infectious viral dose in the serum. Interestingly, both the SMB and original serum had identical C_t_ values of 29, however, only the SMB demonstrated sufficient infectious viral load to infect C6/36 cells and mosquitoes. Further studies are required to ascertain adaptive genetic and phenotypic characteristics of ZIKV in respective mammalian and arthropod hosts. Importantly, given that relatively few ZIKV isolates that have been recovered to date and the inherent constraints in importing ZIKV strains into many countries for diagnostic assay development and research, the successful isolation of TS17-2016 will be essential for continued investigations of ZIKV transmission and pathogenicity.

## Correspondence

Alyssa Pyke: Alyssa.Pyke@health.qld.gov.au

## Competing Interests

The authors have declared that there are no competing interests.

## Data Availability Statement

All relevant data has been disclosed within the manuscript.
